# Modified surgical procedure of corpus callosotomy: rostral corpus callosotomy via the transfrontal approach in dogs

**DOI:** 10.3389/fvets.2025.1649816

**Published:** 2025-08-15

**Authors:** Daisuke Hasegawa, Rikako Asada, Takayuki Miura

**Affiliations:** ^1^Laboratory of Veterinary Clinical Neurology, Graduate School of Nippon Veterinary and Life Science University, Tokyo, Japan; ^2^Veterinary Medical Teaching Hospital, Nippon Veterinary and Life Science University, Tokyo, Japan

**Keywords:** corpus callosotomy, dog, drug-resistant epilepsy, epilepsy, epilepsy surgery, generalized seizures

## Abstract

Corpus callosotomy (CC) is a surgical procedure for palliative epilepsy surgery targeting generalized seizures. In humans, total CC (TCC) is primarily performed in pediatric patients, whereas anterior CC is typically performed in adult patients to avoid postoperative disconnection (split-brain) syndrome, even though the antiseizure effect is inferior to TCC. In dogs, TCC may be more favorable; however, approaching and dividing the rostral part of the corpus callosum (genu) through a previously described bilateral rostrotentorial (dorsal) approach is challenging, particularly in meso- and dolichocephalic and/or large-breed dogs. This approach also risks damaging the rostral cerebral arteries that run along the rostral edge of the genu. Based on our experience, approaching and dividing the genu is easier, safer, and more reliable using the transfrontal approach. This report introduces the rostral CC (RCC) procedure via the transfrontal approach and presents three cases that underwent either transfrontal RCC combined with the dorsal approach to complete TCC or standalone RCC. Although the antiseizure efficacy of RCC alone remains unclear in dogs, this procedure may be useful for completing TCC.

## 1 Introduction

Corpus callosotomy (CC), a longitudinal division of the corpus callosum, the most developed commissural fibers connecting both cerebral hemispheres of the brain, is one of the common and established surgical procedures of palliative epilepsy surgery for human patients with drug-resistant generalized epilepsy (1, 2). As expected, CC interrupts the propagation of seizure activity from one hemisphere to the other, thereby preventing focal seizures from evolving to generalized seizures (i.e., secondarily generalization). In addition, CC disrupts the synchronization of epileptiform discharges and seizure activities between both hemispheres in cases of generalized (bilateral synchronized) seizures. However, since CC is a palliative surgery that does not remove the epileptogenic focus, and seizure propagation may also occur through other pathways such as anterior/posterior commissures, thalamus, and midbrain, it does not always achieve complete seizure freedom.

Total CC (TCC) (or complete CC) – the complete longitudinal division of the entire corpus callosum from the genu (rostral edge) to the splenium (caudal edge) – is generally applied to pediatric patients with drug-resistant epilepsy (DRE), especially those characterized by drop attacks (atonic seizures and myoclonic seizures), such as Lennox–Gastaut syndrome. In adults, on the other hand, partial CC, particularly anterior CC (ACC), which divides the anterior half to four-fifths of the corpus callosum, is commonly performed to avoid postoperative disconnection syndrome, also known as ‘split-brain’ syndrome. Disconnection syndrome occurs more severely in adults with a fully developed and mature brain, whereas the brains of children are immature and may develop compensatory, even after CC.

In 1995, Bagley et al. (3) reported the CC procedure via a bilateral rostrotentorial craniotomy (dorsal approach) in six normal Beagle dogs. Although the intent was to completely divide the corpus callosum (TCC) from the genu (rostral end) to the splenium (caudal end), the genu remained intact in three of the six dogs on postmortem examination. The authors stated in their report that “*division of the callosum was easier caudally and more difficult rostrally*.” About two decades later, we performed CC over 10 cases with DRE, of which the first three cases had been reported (4). In our experience, we have also found that division of the genu via a dorsal approach is highly challenging and carries a risk of injuring the rostral cerebral artery (RCA), especially in mesocephalic and dolichocephalic breeds. This difficulty arises from the distance and direction required to access the genu from the surface of the surgical window (the position of craniotomy), as well as the presence of structures that obstruct access to the genu, such as the confluences of dorsal cerebral veins (DCVs) with the dorsal sagittal sinus (DSS) on the dura and the adhesion of the bilateral cingulate gyri beneath the falx (described in sections 2 and 3.1). In addition, some large-breed dogs have a markedly elevated external sagittal crest attached with massive temporal muscles, making bilateral rostrotentorial craniotomy extremely time-consuming and technically demanding. Therefore, modifications to the procedure are required to make dividing the genu easier and safer.

In this report, we describe the procedure for rostral CC (RCC) via the transfrontal approach, developed based on our experience with CC in dogs. We also present three cases in which this procedure was applied. We believe that this modification is useful for completing TCC or performing standalone RCC in dogs.

## 2 Anatomical knowledge

**Figure 1:**
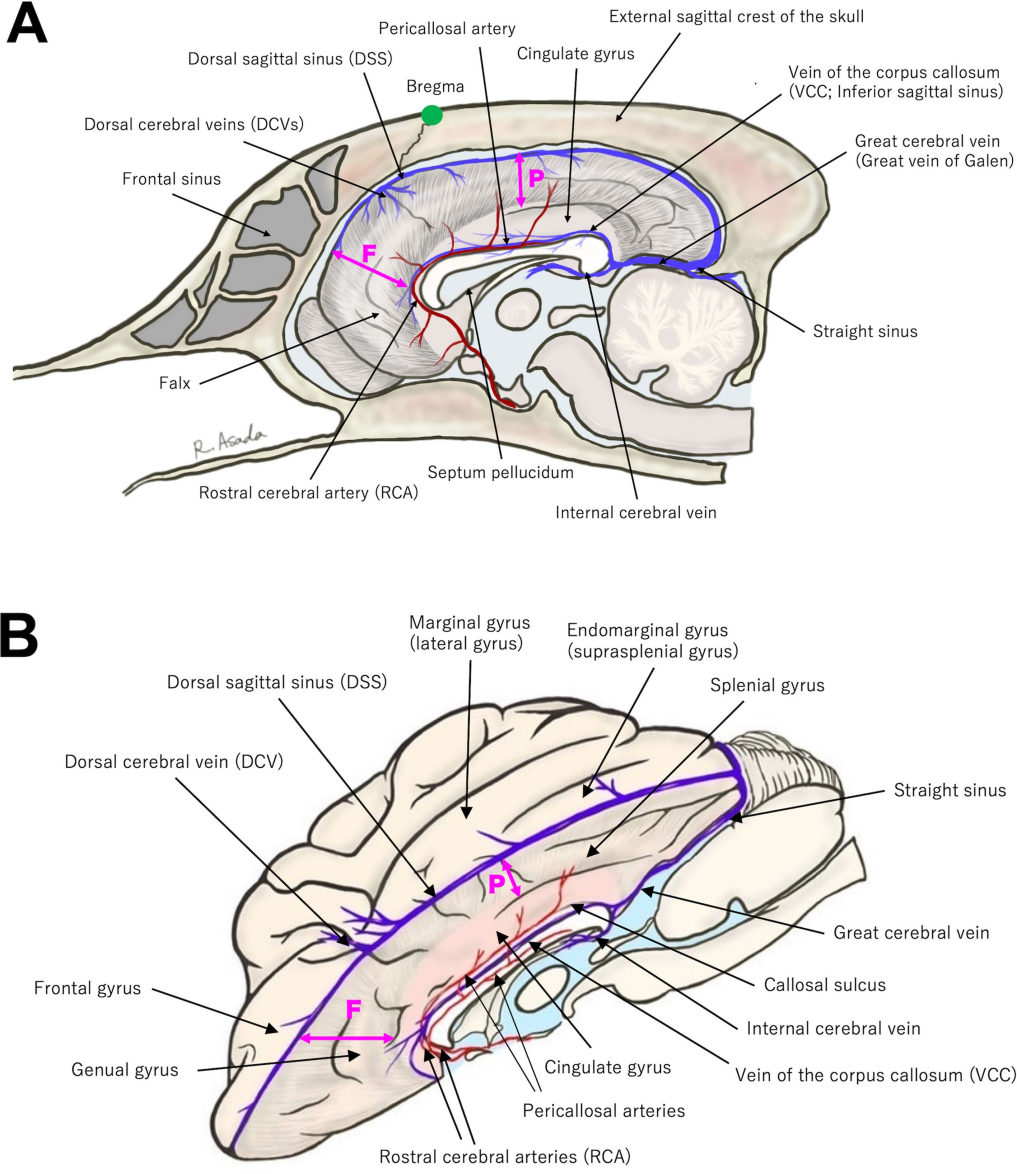
**(A)** Midline sagittal and **(B)** slightly oblique dorsal view (with the left hemisphere removed) illustrations highlighting key anatomical landmarks relevant to corpus callosotomy. Refer to the main text for details. The position of the bregma is indicated by a green dot in **(A)**. The bold double-headed arrows labeled F and P represent the depth of the falx; the frontal portion (F) of the falx is deeper than the parietal portion (P).

To perform CC, the surgeon must have a thorough understanding of the anatomy surrounding the corpus callosum. Figure 1 illustrates the key anatomical structures essential for performing CC.

When performing bilateral rostrotentorial craniotomy, the presence of a prominently elevated external sagittal crest of the skull with massive temporal muscles in some large-breed meso−/dolichocephalic dogs is considerable. It not only prolongs craniotomy but also hinders the careful operation required to preserve the underlying DSS. Moreover, when the procedure is performed by a single surgeon, such a demanding craniotomy may adversely affect the precision of subsequent microsurgical procedures. Next, when determining the craniotomy site, attention should be paid to the positions of the bregma and the confluences of DCVs with DSS. Although there may be some interindividual variation, these structures are located in close proximity, and it is advisable to avoid performing a bone cut at the bregma. The clinical importance of the confluences of DCVs with DSS is described later (section 3.1). On the other hand, the anatomy of the frontal sinus and the technique of transfrontal craniotomy are well-known to veterinary neurosurgeons.

Regarding intracranial structures, DSS runs along the midline surface of the dura, extending from the rostral edge of the olfactory area to the caudal end of the occipital lobe, receiving venous drainage from the cerebral hemispheres via DCVs at several points before terminating at the confluence of the sinuses (junction with straight sinus and tranverse sinuses). The falx extends vertically between the two hemispheres directly beneath DSS. As shown in Figure 1, the falx is relatively deep (broad) in the frontal region but becomes shallow (narrow) in the parietal region, where it is absent at the level of the cingulate gyrus. As a result, the cingulate gyri of both hemispheres are tightly adhered to each other by the pia mater.

Along the ventral margin of the cingulate gyri, the pericallosal arteries run parallel to each other on either side of the midline, with the vein of the corpus callosum running between them over the corpus callosum (Figure 1). The pericallosal arteries, which are extensions of RCAs, provide vascular supply to the medial aspects of both hemispheres. RCAs arise from the rostral portion of the arterial circle of the brain and course dorsocaudally along the rostral surface of the genu of the corpus callosum, continuing as pericallosal arteries. The vein of the corpus callosum, referred to as the inferior sagittal sinus in humans, runs along the midline of the dorsal surface of the corpus callosum, extending from the ventral edge of the falx in the frontal region to the caudal end of the corpus callosum (splenium). It ultimately joins the internal cerebral veins ascending from the ventral side of the corpus callosum and continues as the great cerebral vein (vein of Galen), which courses between the occipital lobes.

The gyri and sulci of the cerebral cortex surrounding the corpus callosum, i.e., medial aspect of the hemisphere, should be recognized; however, the specific functions and/or dysfunctions of these cortical areas in dogs remain unclear, and no clinical signs other than epileptic seizures have occurred when they are damaged. For the dorsal approach, the margin of one hemisphere along the longitudinal fissure, overlying the middle to caudal part of the corpus callosum, consists of the marginal and endomarginal gyri (also referred to as the lateral and suprasplenial gyri). On the medial aspect of the longitudinal fissure, from the dorsal surface of the marginal (suprasplenial) gyrus toward ventrally to the corpus callosum, the following structures are aligned: the suprasplenial sulcus, splenial gyrus, splenial sulcus, cingulate gyrus, and the callosal sulcus, which lies between the corpus callosum and the cingulate gyrus. The aforementioned pericallosal artery runs within the callosal sulcus. From the frontal view (for the transfrontal approach to the genu of the corpus callosum), the frontal and medial surface of the frontal lobe is the frontal gyrus. On the medial aspect of the longitudinal fissure, from the surface of the frontal gyrus toward caudally to the genu, the structures are arranged as follows: the ectogenual sulcus, genual gyrus, genual sulcus, and the rostral part of the cingulate gyrus. As mentioned above, RCA ascends over the surface of the genu and courses along the ventral edge of the cingulate gyrus (within the callosal sulcus), continuing as the pericallosal artery.

Finally, although the terms have been used repeatedly throughout the previous text, the corpus callosum is anatomically divided into three parts: the genu (rostral part), body (middle part), and splenium (caudal part). The septum pellucidum and the third ventricle are located ventral to the genu and body, respectively. Hippocampal commissure, also known as the commissure of fornix, is located beneath the corpus callosum. However, it is difficult to clearly distinguish these structures during surgery. Therefore, at present, our TCC also means complete division of these commissures.

## 3 Methods (surgical procedures)

### 3.1 Previously described corpus callosotomy via the bilateral rostrotentorial craniotomy (dorsal approach)

The surgical procedure of CC, aiming for TCC via a bilateral rostrotentorial craniotomy, has been described in the papers by Bagley et al. (3) and Asada et al. (4). Although the procedure of CC is summarized below, the authors strongly recommend a thorough and careful referencing of the two aforementioned papers.

**Figure 2:**
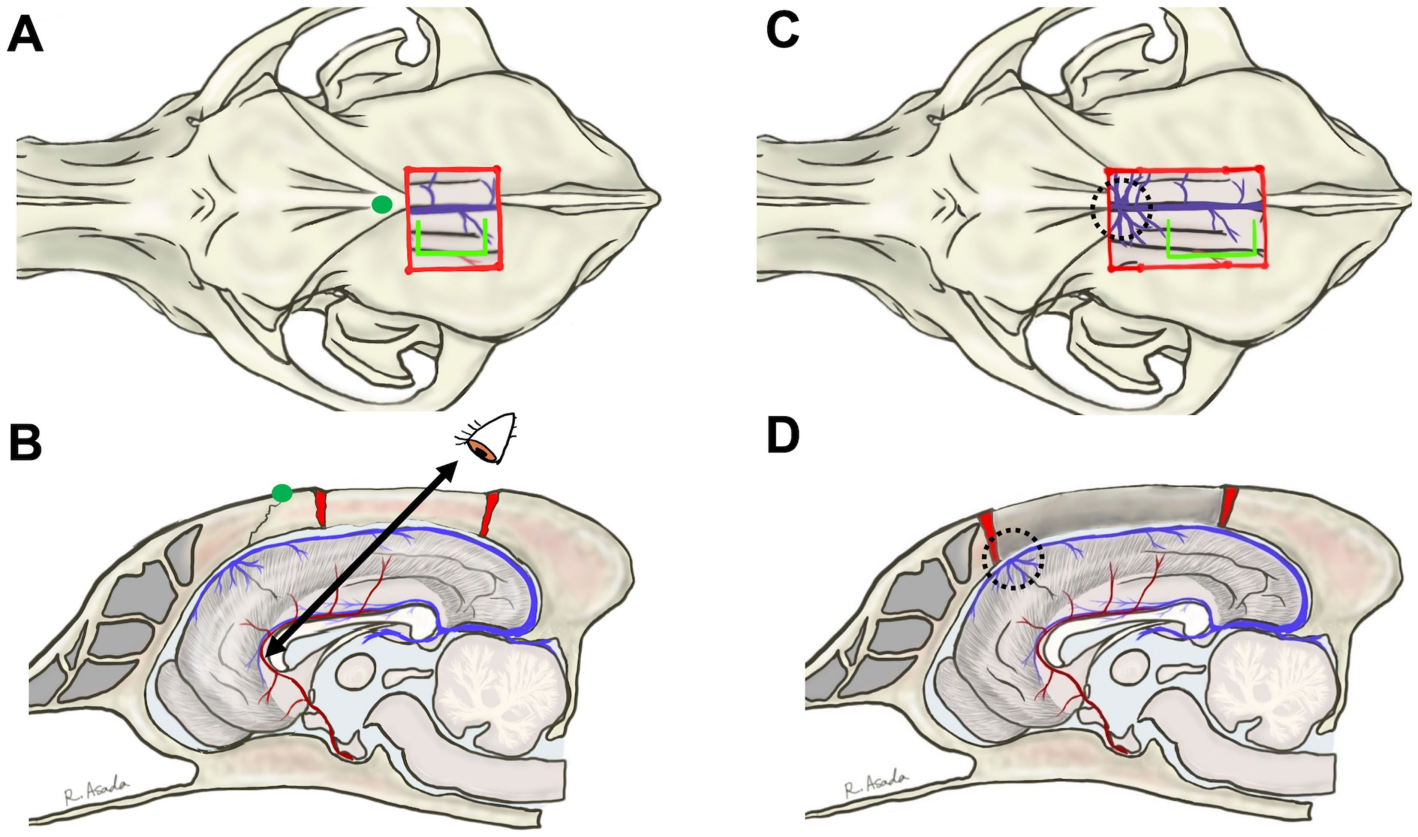
The previously described bilateral rostrotentorial craniotomy and surgical view for dorsally approaching the genu. Dorsal and sagittal views are illustrated for craniotomy caudal to bregma **(A,B)** or beyond bregma **(C,D)**. The position of the bregma is indicated by a green dot in **(A,B)**. In **(A,B)**, the distance to the genu is too large to allow for good intraoperative visibility. In **(C,D)**, the confluence of the dorsal cerebral veins with the dorsal sagittal sinus (dotted circles, and see also Figure 3A) obstructs access to the genu. In both dorsal approaches, access to the genu must be from a dorsocaudal direction, making it difficult to see the rostral surface of the genu and the rostral cerebral artery. Blight lime green lines in **(A,C)** indicate the line of a dorsal sagittal sinus-based U-shaped durotomy, where the rostral edge of the incision is positioned caudal to the confluence.

**Figure 3:**
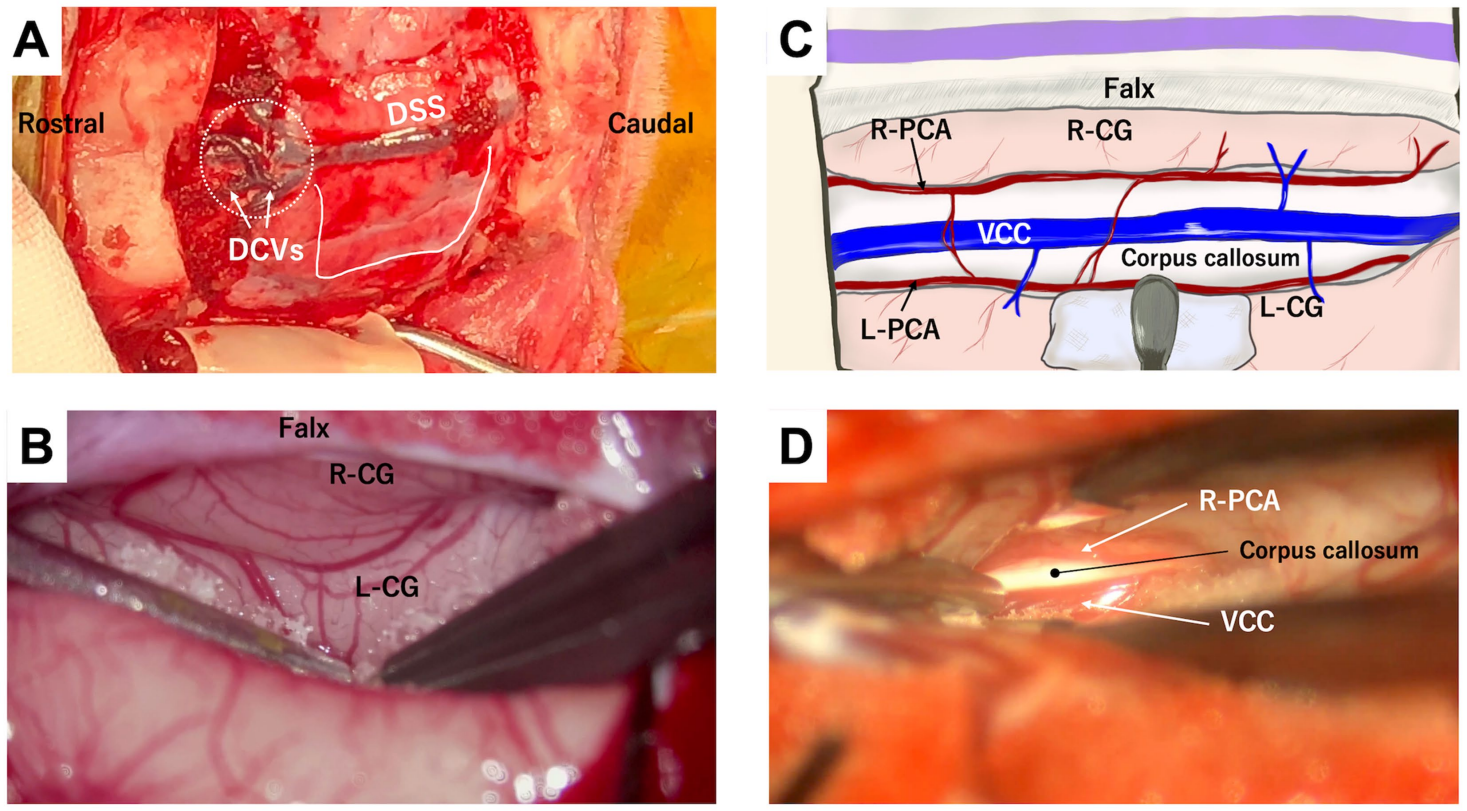
Intraoperative views of rostrotentorial (dorsal) approach to the corpus callosum. **(A)** Photograph showing the surgical window following bilateral rostrotentorial craniotomy extending beyond the bregma. Dorsal sagittal sinus (DSS) is visible along the midline, with some thick dorsal cerebral veins (DCVs) draining into it. Dotted circle indicates the location of the confluence of DCVs with DSS, which should be avoided to prevent damage. U-shaped white line shows the durotomy line, with its rostral edge positioned caudal to the confluence. **(B)** Microscopic photograph showing retraction of the left hemisphere away from the falx. Beneath the falx, the right (R-CG) and the left (L-CG) cingulate gyri are tightly adhered to each other, making it challenging to identify the midline and time-consuming to separate them. **(C,D)** Show a conceptual illustration and a microscopic photograph, respectively, after separating the cingulate gyri. The vein of the corpus callosum (VCC) runs along the midline above the corpus callosum, flanked by the parallelly running left (L-PCA) and right (R-PCA) pericallosal arteries. These arteries typically lie beneath the cingulate gyrus within the callosal sulci; therefore, the left pericallosal artery (L-PCA) is not visible in **(D)**.

A bilateral rostrotentorial craniotomy with one side opened more widely – approximately 2 cm from the midline on the left side for a right-handed operator and about 1 cm on the opposite side – is performed either caudal to the bregma (Figures 2A,B) or extending beyond the bregma (Figures 2C,D, 3A). The rostrocaudal length of these craniotomies is planned to cover the entire length of the corpus callosum, which varies depending on the individual or skull type, typically ranging from 3 to 5 cm. As mentioned in the section of Anatomical Knowledge, the position of the bregma and/or confluence of DCVs with DSS should be confirmed with preoperative imaging (contrast-enhanced MRI or CT). The dura is cut in a U-shape, based on the midline (DSS), and reflected to the opposite side. During this process, small cerebral veins draining into the DSS are cauterized and cut between the cortex and the reflected dura using bipolar cautery. However, typically, two or three relatively thick DCVs drain into the DSS over the cruciate sulcus (Figures 3C,D; dotted circle) and should be preserved. If these DCVs are damaged or cauterized, severe cerebral edema may develop in the hemisphere, disrupting the callosotomy procedure and occasionally leading to tentorial herniation. This complication makes CC more challenging and may result in severe postoperative complications. As a result, the rostral edge of the durotomy is positioned caudal to this confluence.

Then, one hemisphere is gently retracted laterally from the falx, with a piece of neurosurgical pattie placed on its surface for protection, using a thin spatula, fine brain retractor, or the shaft of bipolar forceps. Maintaining the retracted position of the cortex is technically challenging. Therefore, the authors insert multiple neurosurgical patties into the longitudinal fissure to create and sustain sufficient space for microsurgical manipulation described below; the position and amount of patties are adjusted as the dissection advances. The marginal (lateral) and suprasplenial gyri are easily separated due to the presence of the falx. However, the falx in the parietal region is shallow (Figure 1), and the bilateral cingulate gyri beneath it often adhere to each other. Careful and blunt longitudinal separation of the adhesive cingulate gyri is the most time-consuming and challenging aspect of the TCC procedure.

Beneath the separated cingulate gyrus, a pair of longitudinally running pericallosal arteries (continuation of RCAs), the vein of the corpus callosum, and the underlying corpus callosum are visible. A few thin bridge vessels between the cortex and the vein of the corpus callosum, as well as between bilateral pericallosal arteries, can be cauterized, cut, and separated to either side. Mobilizing these vessels laterally to either side allows exposure of the underlying portion of the corpus callosum. During this process, short, fine-tip bipolar forceps are strongly recommended. Because the corpus callosum is located deeply, the outer surface of the tips of standard bipolar forceps may contact structures, such as the cingulate gyrus or vein of the corpus callosum, potentially leading to inadvertent coagulation. An initial incision is made on the body of the corpus callosum using a micro-knife or a bipolar cautery. When penetrating the corpus callosum, cerebrospinal fluid (CSF) flows from the cavity of the septum pellucidum and/or the third ventricle. Then, dividing the corpus callosum proceeds caudally to the splenium and rostrally to the genu.

Completion of the splenium division is confirmed by observing the site where the vein of the corpus callosum and the internal cerebral vein merge to form the great cerebral vein. While dividing the body, the choroid plexus, the course of the internal cerebral vein, and the septum pellucidum are observed ventral to the corpus callosum. However, as the division advances rostrally, the genu becomes too distant to access through the surgical window created by the rostrotentorial craniotomy (Figure 3B). Furthermore, preserving the confluence of DCVs and DSS occasionally obstructs the visibility of the genu (Figure 3D). Therefore, the genu requires division from a deep dorsocaudal aspect, making the RCAs on the rostral edge of the genu invisible, which can result in an incomplete division of the corpus callosum (see also Section 4.1 Case 1) and/or a risk of damaging the RCAs.

### 3.2 Rostral corpus callosotomy via the transfrontal approach

Instead of using a dorsal approach, this ‘Methods’ report describes the procedure for dividing the genu via the transfrontal approach.

**Figure 4:**
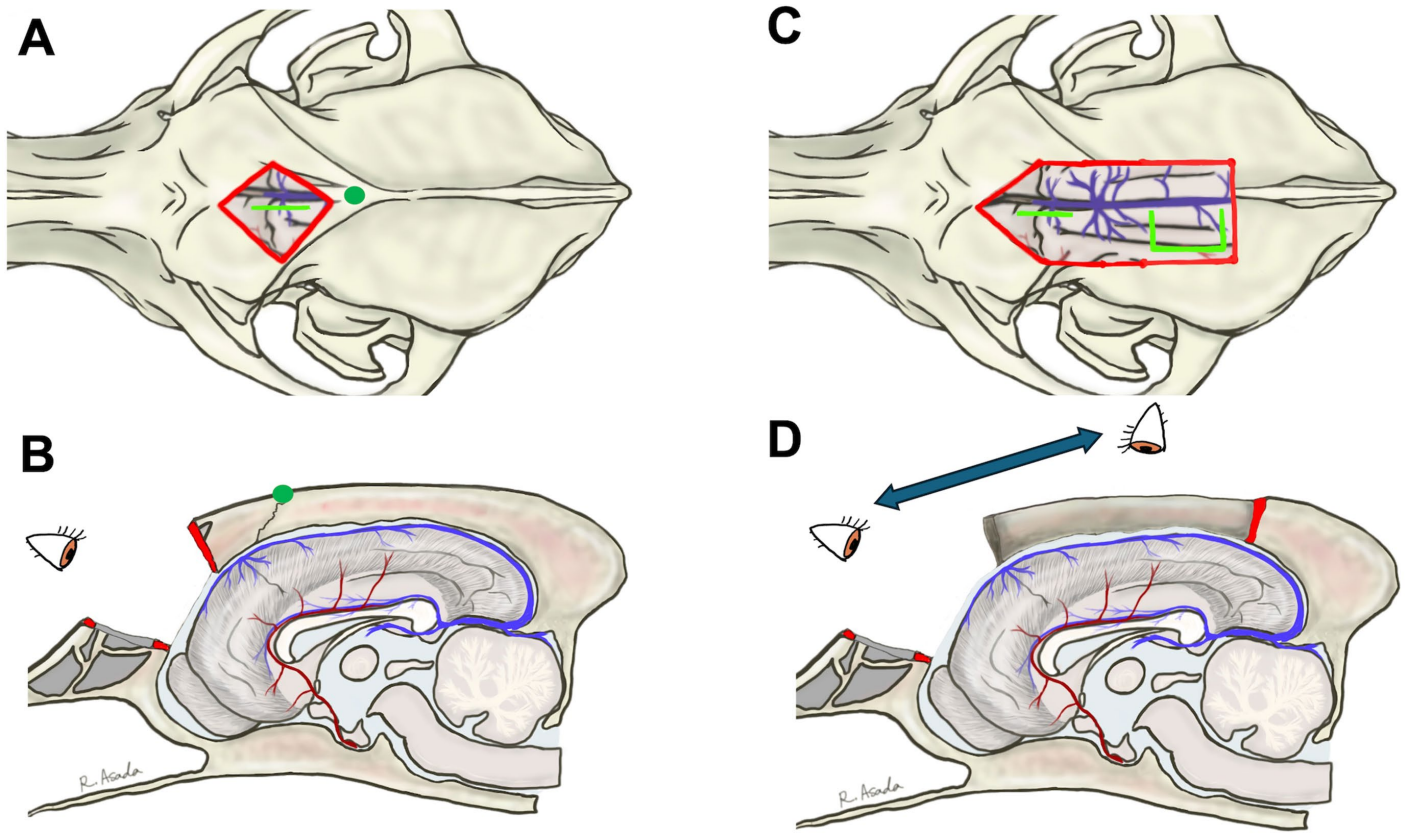
Bilateral diamond-shaped transfrontal craniotomy for rostral corpus callosotomy alone **(A,B)** and a combination of transfrontal and rostrotentorial craniotomy **(C,D)** for one-stage total corpus callosotomy. Blight lime lines in **(A,C)** are the lines of the durotomy. In the transfrontal approach, the surgeon can directly visualize the genu and rostral cerebral arteries **(B,D)**. When performing a one-stage total callosotomy, the surgeon can change their position between the division of the rostral part and the caudal part of the corpus callosum **(D)**.

For standalone RCC or a combination of transfrontal and rostrotentorial craniotomy for TCC, a bilateral diamond-shaped transfrontal craniotomy is recommended (Figures 4A,B). Alternatively, if TCC is planned through a single surgical window, an extended transfrontal to rostrotentorial craniotomy can be made (Figures 4C,D). While a unilateral transfrontal craniotomy might be feasible, since the approach to the genu is conducted from a single hemisphere, the remaining frontal bone obstructs the intracranial and microscopic operation. After thoroughly irrigating and disinfecting (using povidone-iodine) the frontal sinus, the internal table is removed to expose the dura.

**Figure 5:**
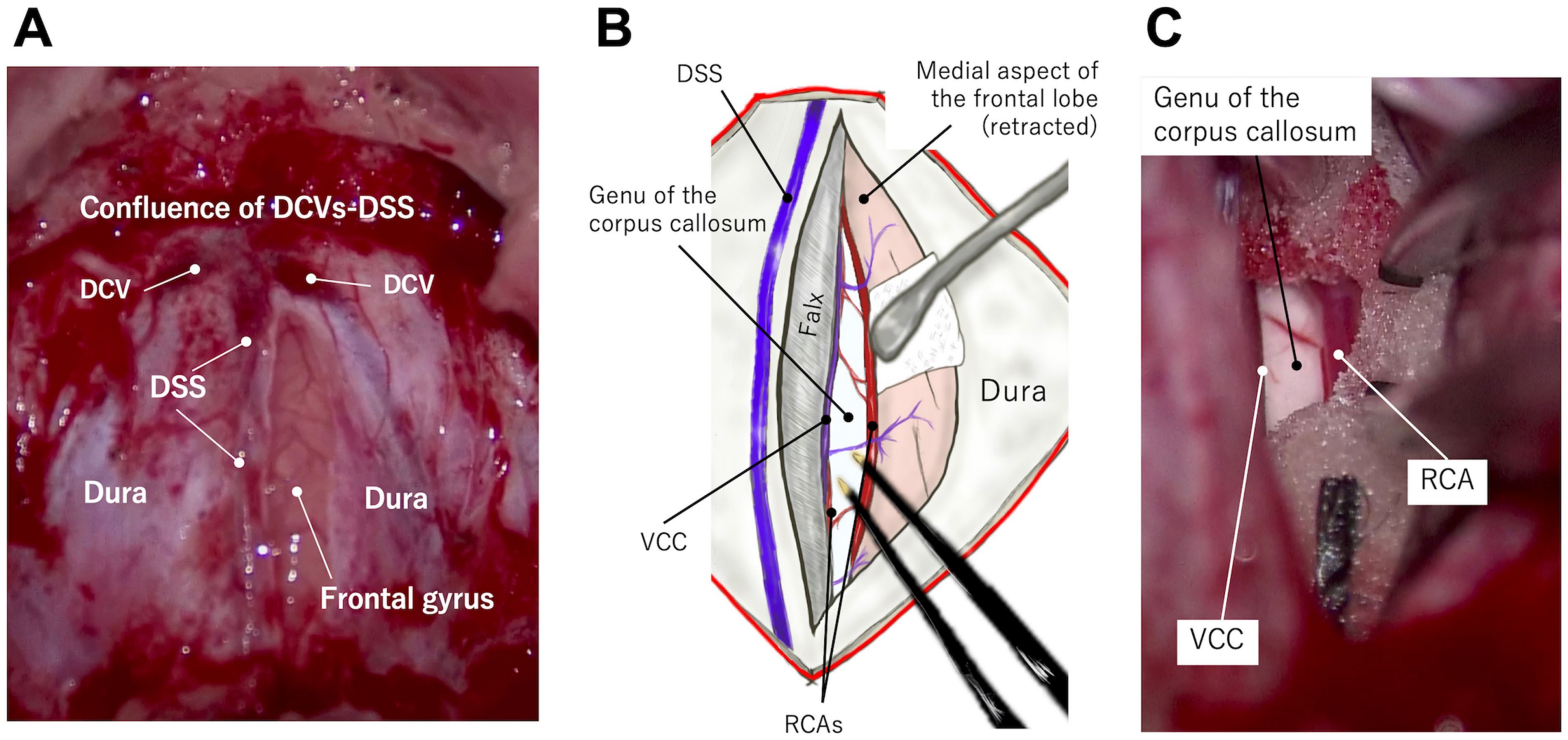
Microscopic photographs **(A,C)** and a conceptual illustration **(B)** demonstrating access to the genu via the transfrontal approach. A longitudinal straight durotomy is made immediately lateral to the dorsal sagittal sinus (DSS) and rostral (ventral) to the confluence of dorsal cerebral veins (DCVs) with DSS **(A)**. When one frontal lobe is retracted laterally **(B,C)**, the vein of the corpus callosum (VCC) and rostral cerebral artery (RCA) are visible at the ventral (deep) edge of the falx and the frontal lobe, respectively. The genu lies just beneath these vessels. See also Supplementary Video S1.

The dura is incised longitudinally, immediately lateral to the DSS and rostral to its confluence with DCVs (Figures 4A,C, 5; Supplementary Video S1). After applying a neurosurgical patty, one frontal lobe is gently retracted with a spatula or the shaft of bipolar cautery forceps. In this region, unlike in the parietal region, the falx extends deeply enough to preserve the contralateral cortex, and there is minimal adhesion between the cingulate gyri. Therefore, the vein of the corpus callosum can be observed deep within the retracted medial surface of the frontal cortex along the ventral edge of the falx. The left and right RCAs (running almost parallel) are further ventral, leading to the genu of the corpus callosum, which is visible as a bright white structure (Figure 5; Supplementary Video S1). There are a few thin vessels between the cortex and the vein of the corpus callosum, and between the paired RCAs, which can be cauterized and cut. This procedure allows one cortex and RCA to be loosened, providing good visibility and space for dividing the genu.

The genu is then divided, as described above. When penetrating the corpus callosum, CSF flows from the cavity of the septum pellucidum, which appears as a longitudinal groove (Supplementary Video S1). The division of the genu proceeds ventrally until the callosum is completely divided and then dorsocaudally as far as possible (see also Section 4.3 Case 3). For TCC, if the body is already divided via the dorsal approach, the rostral division is extended to connect that division site (see also Section 4.1 Case 1). Repositioning of the surgeon and microscope is necessary when switching from the transfrontal approach for RCC to the dorsal approach for dividing the caudal (body and splenium) part (Figure 4D). After finishing RCC (or TCC), hemostasis in the intracranial surgical field is confirmed, and the dura is closed in a watertight manner using 5–0 or 6–0 absorbable monofilament with simple interrupted sutures. The bone flap is then replaced and fixed using polypropylene sutures, with 2 or 3 sutures typically placed along each edge. If unacceptable gaps remain between the bone flap and the skull, they should be filled with bone wax or polymethyl methacrylate.

## 4 Results (case presentation)

This section introduces three cases in which RCC was conducted via the transfrontal approach with a 1-year follow-up. A summary of these cases is shown in Table 1. These cases are part of our research project titled “Development of Epilepsy Surgery in Small Animal Veterinary Medicine,” which was funded by the grants of KAKENHI and PMAC and approved by the Ethical Committee for Clinical Studies of the Veterinary Medical Teaching Hospital of Nippon Veterinary and Life Science University (VMTH-NVLU). Details of the clinical trial of epilepsy surgery, including the case selection criteria for CC, are shown in Supplementary File S1. Informed consent was obtained from each dog’s owner following an in-person consultation, during which the purpose and design of the clinical trial, sources of funding, potential benefits and risks of the CC and alternative surgical techniques, the surgical procedure itself (including a transfrontal modification), and follow-up schedules were thoroughly explained. The owner of each dog signed a written consent form. The overall results and long-term outcomes of the clinical trial will be published separately. This paper reports only three cases from a clinical trial in which a transfrontal rostral CC was applied.

**Table 1:** Summary of three cases that underwent rostral corpus callosotomy using the transfrontal approach.

	Case 1	Case 2	Case 3
Breed	Beagle	Whippet	Golden Retriever
Sex (at referral)	Neutered male	Intact female	Neutered female
Age (at referral)	3 y.o.	1 y.o.	1 y 11 m.o.
Age at the initial sz	1 y.o.	4 m.o.	1 y.o.
Epilepsy type	Idiopathic	Structural (congenital)	Idiopathic
Seizure types	FBS GTCS	FBS GTCS	AtS GTCS
Presence of CS/SE	+/+	+/+	+/+
ASMs	PB, KBr, ZNS, LEV, PGB	PB, KBr, ZNS, LEV	ZNS, LEV, KBr, PB
Preop. seizure frequency –ave. of preop. counted months (baseline)	Total: 7.3 sz/m FBS: 1.7 sz/m GTCS: 6.0 sz/m	Total: 11.2 sz/m FBS: 7.6 sz/m GTCS: 3.5 sz/m	Total: 36.3 sz/m AtS: 27.7 sz/m GTCS: 8.6 sz/m
Preop. seizure day frequency	6.8 szd/m	8.8 szd/m	8.3 szd/m
Surgical procedures	Two-stage total CC 1st op: 2/3 caudal CC 2nd op: 1/3 rostral CC	One-stage total CC combining transfrontal and dorsal approaches	Standalone rostral CC
Postop. seizure frequency – ave. of postop. 1-year (reduction rate)	Total: 4.4 sz/m (40%R) FBS: 2.3 sz/m (+35%) GTCS: 2.1 sz/m (65%R)	Total: 17.0 sz/m (+52%) FBS: 10.0 sz/m (+32%) LTS: 7.0 sz/m (new) GTCS: 0 sz/m (100%R)	Total: 39.6 sz/m (+9%) AtS: 0 sz/m (100%R) FMBS: 32 sz/m (new) GTCS: 8.6 sz/m (±0%)
Postop. seizure day frequency	3.8 szd/m (44%R)	9.6 szd/m (+9%)	4.3 szd/m (52%R)
Post-2nd-op. seizure frequency – ave. of post-2nd-op. 1-year (reduction rate)	Total: 3.4 sz/m (53%R) FBS: 0.4 sz/m (76%R) GTCS: 2.9 sz/m (52%R)	NA	NA
Postop. complications	None	Temporary (<1 week) left forebrain signs	None

FBS, focal behavioral seizures; GTCS, generalized tonic–clonic seizures; LTS, lateralized tonic seizures; AtS, atonic seizures; FMBS, focal motor and behavioral seizures; CS, cluster seizures; SE, status epilepticus; ASM, antiseizure medication; PB, phenobarbital; KBr, potassium bromide; ZNS, zonisamide; LEV, levetiracetam; PGB, pregabalin; GBP, gabapentin; IMP, imepitoin; sz/m, seizures/month; szd/m, seizure days/month; CC, corpus callosotomy. Reduction rate is calculated as (baseline – postoperative) / baseline × 100.

### 4.1 Case 1

A 3-year-old, neutered male Beagle dog with idiopathic epilepsy (IVETF Tier III level), which had focal behavioral seizures and generalized tonic–clonic seizures (GTCSs) resistant to the maximum doses (or serum concentrations) of five antiseizure medications (ASMs), was referred to VMTH-NVLU for consideration of epilepsy surgery. The 6-month average seizure frequencies at the time of referral (baseline) were 7.3 seizures/month (sz/m) and 6.8 seizure days/month (szd/m) for all seizure types, including 6.0 sz/m for GTCS and 1.7 sz/m for focal behavioral seizures. On the scalp EEG, bilateral synchronized epileptiform discharges (spikes and sharp waves) and their bursts were observed without an apparent localized focus. As a result of the judgment for indicating epilepsy surgery, CC and vagus nerve stimulation (VNS) were proposed as treatment options. After receiving a detailed explanation of each procedure, the dog’s owner elected to proceed with the CC.

**Figure 6:**
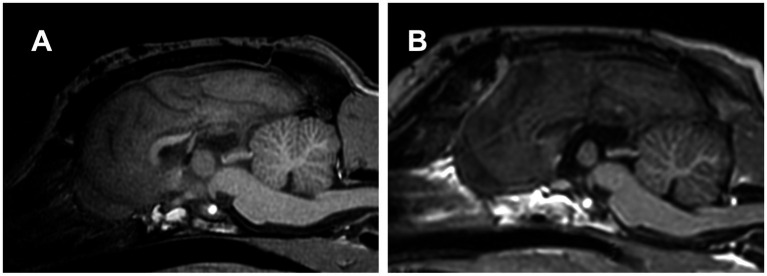
**(A)** Postoperative midline sagittal T1-weighted image of Case 1 (a Beagle; mesocephalic breed) after the first surgery, in which total corpus callosotomy was attempted via a dorsal approach. However, the rostral part of the corpus callosum remained intact, resulting in caudal corpus callosotomy only. **(B)** Postoperative image in the same plane after the second surgery, performed 1 year later as a two-stage procedure to complete total corpus callosotomy via the transfrontal approach. The previously remaining rostral part was completely divided.

To perform TCC, the previously reported (3, 4) and aforementioned CC procedure, i.e., the dorsal approach, was conducted, as shown in Figures 3C,D. Although the body and splenium were successfully divided, the division of the rostral part of the callosum was extremely challenging due to its distance from the surgical window and the presence of the confluence of DCVs and DSS. Therefore, we abandoned completion of TCC during this surgery and decided to follow the postoperative course for 1 year as a partial (caudal) CC (Figure 6A). During this 1-year follow-up period after caudal CC, seizure frequency did not show marked improvement overall. Although >50% seizure reduction was recorded only in GTCS, there was a 4.4 sz/m (42% reduction) and a 3.8 szd/m (44% reduction) for all seizure types, including 2.1 sz/m (65% reduction) for GTCS and 2.3 sz/m (35% increase) for focal behavioral seizures.

Consequently, after thorough discussion and obtaining informed consent from the owners, RCC via the transfrontal approach was performed as a two-stage surgery to complete the TCC, as described above, without any intraoperative difficulty. Thus, TCC was successfully achieved through two separate surgeries performed 1 year apart (Figure 6B). There were no postoperative complications after the second surgery (RCC). During a 1-year follow-up after completing TCC (i.e., following the second surgery), the seizure frequency improved further. The average seizure frequency for all seizure types was reduced to 3.4 sz/m, reflecting a 53% reduction from the preoperative baseline and an additional 23% reduction compared to that after the first surgery. Similarly, averaged seizure days decreased to 3.25 szd/m, corresponding to a 52% reduction from baseline and an additional 14% reduction from the first surgery. For GTCS, the seizure frequency was 2.9 sz/m (60% reduction from baseline, but a 38% increase compared to the first surgery). In contrast, focal behavioral seizures decreased markedly to 0.4 sz/m (76% reduction from baseline and 83% reduction compared to the first surgery).

### 4.2 Case 2

A 1-year-old, intact female Whippet dog with structural generalized epilepsy probably caused by abnormal gyration in the bilateral fronto-parietal cortex (from structural MRI) and resistant to sufficient doses or serum concentrations of four ASMs was referred to VMTH-NVLU for CC. The seizure types were focal behavioral seizures and GTCSs, according to the ictal videos. Averaged monthly seizure frequency and seizure days during the 6 months prior referral were 11.2 sz/m and 8.8 szd/m for all seizure types, including 3.5 sz/m for GTCS and 7.6 sz/m for focal behavioral seizures. Interictal scalp EEG revealed bilaterally synchronized epileptiform discharges, predominantly in the bilateral fronto-parietal regions. After CC and VNS were suggested as treatment options, the owners chose CC.

**Figure 7:**
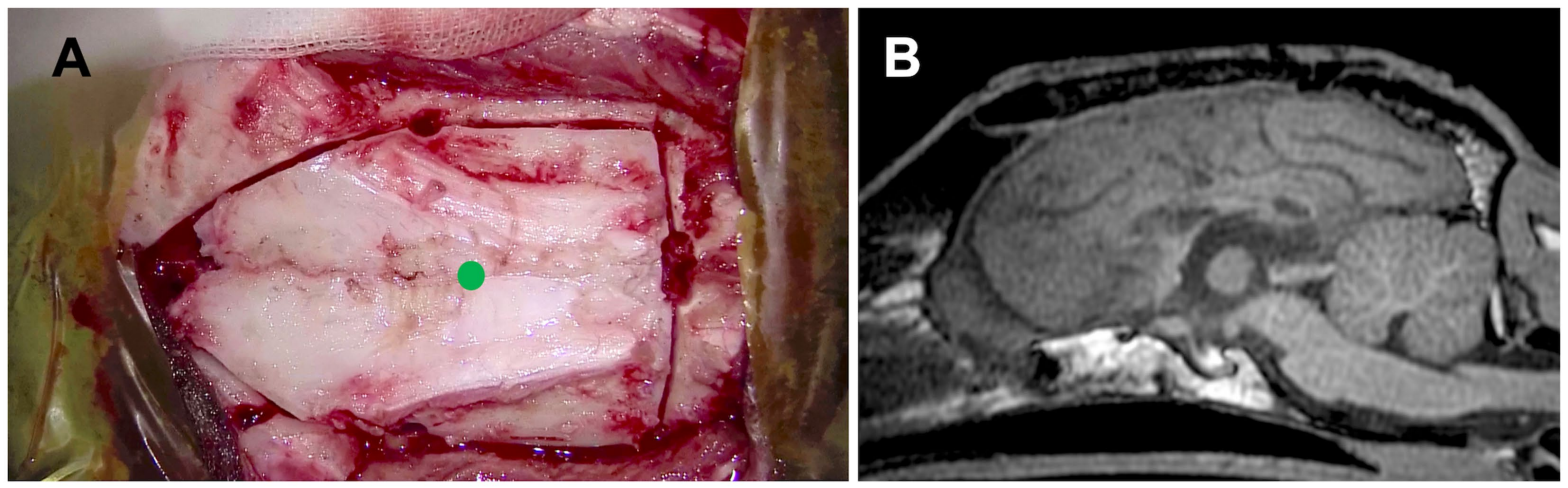
**(A)** Intraoperative photograph showing the extent of the combination of transfrontal and rostrotentorial craniotomy, and **(B)** postoperative midline sagittal T1-weighted image of Case 2 (a Whippet; dolichocephalic breed). In this case, a one-stage total corpus callosotomy was achieved using an extended craniotomy through both frontal and dorsal approaches, with surgeon repositioning, even in a dolichocephalic dog. A green dot in **(A)** indicates the position of the bregma.

Because the Whippet is a dolichocephalic breed and based on the experience with Case 1, a combination of transfrontal and rostrotentorial craniotomy (Figures 4C,D, 7A) was performed to achieve one-stage TCC. Division of the rostral part (genu) of the corpus callosum, i.e., RCC, was first performed via the transfrontal approach. This was followed by the division of the middle (body) and caudal (splenium) parts of the corpus callosum via the dorsal approach, thereby completing the TCC in a single operation (Figure 7B). Postoperatively, mild left forebrain signs –proprioceptive deficit in the right limbs – were observed but resolved within 1 week.

During the postoperative 1-year follow-up period, this case showed disappearance of GTCS (100% reduction), while hemi-lateralized tonic seizures and focal behavioral seizures, which had a very short duration (few seconds), increased and those frequencies were 7.0 sz/m and 10.0 sz/m, respectively. The owners of this case said, “Even if seizures occur, they only last a few seconds, so daily living has become much better for her and for us.” Thus, although the total seizure counts had increased, there was a marked reduction in seizure severity and improved quality of life.

### 4.3 Case 3

A 3-year-old female Golden Retriever with idiopathic epilepsy (Tier III confidence level), presenting drug-resistant GTCS and atonic seizures, was referred to VMTH-NVLU as a candidate for epilepsy surgery. The 3-month-averaged seizure frequency and seizure days were 36.3 sz/m and 8.3 szd/m for all seizure types, including 8.6 sz/m for GTCS and 27.7 sz/m for atonic seizures. Interictal scalp EEG showed bilaterally synchronized spikes and sharp waves, predominantly in the midline frontoparietal region. Although VNS was recommended over CC due to the dog’s head shape, and its owner was informed of the procedures, advantages, and disadvantages of both VNS and CC, respectively, the owner ultimately chose CC because the frequent hospital visits required to adjust the stimulation intensity of VNS were difficult to accommodate within their lifestyle at the time. As mentioned above, the bilateral rostrotentorial craniotomy and dorsal approach to the corpus callosum are challenging in large-breed and mesocephalic dogs with a highly elevated sagittal crest on the skull. Therefore, after a thorough discussion between the owner and our team, we decided to first perform standalone RCC via the transfrontal approach. Then, if improvement was insufficient, a two-stage TCC or reconsideration of the VNS would be planned.

**Figure 8:**
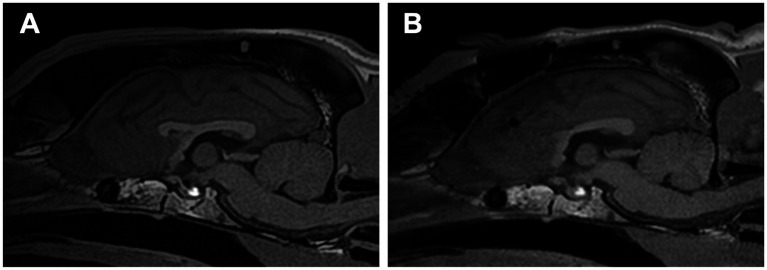
**(A)** Preoperative and **(B)** postoperative midline sagittal T1-weighted images of Case 3 (a Golden Retriever; mesocephalic breed) that underwent standalone rostral corpus callosotomy via the transfrontal approach. Note the prominently elevated sagittal crest on the skull, which is more pronounced than in Cases 1 and 2.

Standalone transfrontal RCC was performed, as described in Section 3.2, without any difficulties or complications (Figure 8). Postoperatively, atonic seizures completely disappeared (100% reduction), and the average number of monthly seizure days decreased by ≥50%. However, GTCSs and newly onset focal motor or behavioral seizures (sudden tonic mouth opening and running) persisted and were often clustered. Postoperative 1-year averaged seizure frequencies and seizure days were 39.6 sz/m (9% increase) and 4.3 szd/m (52% reduction) for all seizure types, including 8.6 sz/m for GTCS and 32 sz/m for focal seizures. In the final 2 months of the postoperative year, the number of days with clustered focal seizures increased. The owner chose VNS over completion of TCC, and VNS was subsequently performed in this case. At 5 months after VNS, the owner reported a marked reduction in GTCS, although brief focal seizures still occurred daily.

## 5 Discussion

Although CC was first reported in dogs over 20 years ago (3), it has not become a commonly performed procedure in veterinary medicine, likely due to technical difficulties associated with the conventional dorsal approach via a bilateral rostrotentorial craniotomy. To the best of our knowledge, this report presents the first description of RCC in dogs, which was devised not as a standalone procedure (as is often the case in adult humans) but as a technique to overcome the challenges of dividing the rostral part (genu) of the corpus callosum in previously reported TCC procedures in dogs (3, 4).

The greatest advantage of the transfrontal approach lies in the enhanced visibility of the RCA and the frontal aspect of the genu, which are difficult or impossible to access via the dorsocaudal (rostrotentorial) approach. The RCA is a critical artery supplying the medial frontal and parietal lobes, including the primary motor and sensory cortices (5). Inadvertent damage to the RCA at the level of the genu may result in extensive iatrogenic ischemia. The transfrontal approach allows for safer division of the genu under direct visualization of the RCA. In addition, the frontal falx is deep, and the paired cingulate gyri are minimally adhered to in this region, facilitating easier interhemispheric access compared to more caudal (parietal and occipital) regions, which require delicate dissection.

As a result, we now routinely perform a combination of rostral (frontal) and caudal (dorsal) callosal division to achieve one-stage TCC in dogs. This modification has made the procedure more feasible and consistent in practice, particularly in mesocephalic and dolichocephalic breeds with prominent sagittal crests and thick temporal musculature, where bilateral rostrotentorial craniotomy can be time consuming and technically challenging.

In human medicine, ACC is often used in adult patients with DRE who have generalized or secondarily generalized focal seizures but are not candidates for resection surgery due to multiple or unidentified foci (1, 2). The rationale for ACC in adults is to reduce the risk of postoperative disconnection (split-brain) syndrome, a complication that can significantly impair quality of life. Most cases of the disconnection syndrome in humans are acute and transient, presenting with symptoms such as aphasia, apathy, hemineglect, hemiparesis, and occasionally alien hand syndrome (1, 2, 6–11). These symptoms typically improve within a few months to years, depending on factors such as the patient’s age (i.e., functional brain maturity), underlying conditions, and the extent of corpus callosum division (ACC or TCC) (6–11). However, in some cases, especially those underwent TCC, long-term neurological sequelae persist permanently (1, 8, 9). In contrast, no cases of “apparent” disconnection syndrome have been reported in dogs undergoing TCC, either in previous reports (3, 4) or in our own experience (Cases 1 and 2 of this report); however, transient mild ataxia and hemiparesis (contralateral to the retracted hemisphere) were observed in most cases, and one case [Case 2 in our previous report (4)] exhibited transient cognitive dysfunction. These signs resolved within a week to a few months, but it remains unclear whether they are consisted with disconnection syndrome. On the other hand, one case underwent standalone RCC (Case 3 in this paper) did not show any postoperative complications.

When comparing ACC and TCC in human medicine, the antiseizure effect is undoubtedly superior in TCC (6–11). Therefore, if seizure control is insufficient after ACC, posterior CC may be added later as a two-stage surgery to achieve TCC (6–8). In dogs with DRE, when feasible, we recommend one-stage TCC, as it offers superior seizure control without the risk of disconnection-related complications. However, in specific breeds with challenging skull morphology, standalone RCC may serve as a reasonable first step. If seizure control is inadequate, a two-stage TCC or alternative treatments such as VNS (12, 13) should be considered – an approach similar to that used in adult humans. While VNS is considered less invasive than CC, CC may present a more practical and accessible treatment option in veterinary medicine at present because VNS devices are extremely expensive, require multiple visits to adjust the stimulation strength, and their therapeutic effects take considerable time to manifest.

Importantly, this report focuses solely on the surgical technique of RCC via the transfrontal approach as a modification to facilitate safer and more effective TCC in dogs. The clinical efficacy of RCC or TCC, including seizure outcomes and quality-of-life assessments, will be addressed in future studies. We hope that this surgical modification will serve as a breakthrough in promoting the adoption of epilepsy surgery in veterinary medicine and will contribute to improving the prognosis of dogs with DRE.

## Data Availability

The original contributions presented in the study are included in the article/Supplementary material, further inquiries can be directed to the corresponding author.

## Supplementary material

Supplementary Video S1 Microsurgical video of a transfrontal rostral corpus callosotomy (edited at 2–4 × speed – recommended playback at 0.5×; low resolution).

Supplementary File S1 Supplementary information on the clinical trial of epilepsy surgery in dogs with drug-resistant epilepsy.
