# Supplementary material for: Modified surgical procedure of corpus callosotomy: rostral corpus callosotomy via the transfrontal approach in dogs
Source: Front Vet Sci. 2025 Aug 15;12:1649816. doi: 10.3389/fvets.2025.1649816 (PMC12396196; doi:10.3389/fvets.2025.1649816)
Supplement: Supplementary File S1 — Supplementary information on the clinical trial of epilepsy surgery in dogs with drug-resistant epilepsy. [file Supplementary_file_1.pdf]

Epilepsy Surgery in Dogs with Drug-Resistant Epilepsy: A Clinical Trial

1. Background, Purpose, and Funding

To establish and propagate epilepsy surgery as an alternative treatment option for dogs with drug-resistant epilepsy, a research project titled "Development of epilepsy surgery in small animal veterinary medicine" by its research team, the "veterinary epilepsy surgery team (VEST)," was launched in 2017 by Professor Daisuke Hasegawa (as the primary investigator) at Nippon Veterinary and Life Science University. The VEST comprised four board-certified veterinary neurology specialists and one veterinary neuropathologist from four institutions, working in collaboration with human medical doctors specializing in epileptology and epilepsy surgery.

Between 2017 and 2021, several studies were initiated with support from KAKENHI (Grants-in-Aid of Scientific Research; grant number 17H01507), including basic studies regarding the methodology for identifying the epileptogenic zone and surgical procedures and the clinical trial of epilepsy surgery in dogs and cats with drug-resistant epilepsy. Since the clinical trial was not completed within the KAKENHI funding period, it continued from 2022 to 2024 with support from the Science Research Promotion Fund by the Promotion and Mutual Aid Corporation for Private Schools of Japan (PMAC).

Regarding the clinical trial (2018–2024), both canine and feline candidates for epilepsy surgery were recruited as described below; however, due to the extremely limited number of candidate cats and the fact that surgery was ultimately performed in only one feline case during the 7-year study period, the clinical trial was effectively conducted in dogs only.

2. Ethics

The clinical trial plan for epilepsy surgery in dogs with drug-resistant epilepsy – including study design, case recruitment and informed consent procedures, presurgical evaluations, surgical techniques, postoperative care, follow-up management, and supporting fee – was reviewed and approved by the Ethical Committee for Clinical Studies of the Veterinary Medical Teaching Hospital of Nippon Veterinary and Life Science University.

3. Case Recruitment and Inclusion Criteria

Candidate cases – canine patients suspected of having drug-resistant epilepsy – were recruited through public announcements on the Teaching Hospital's website and through promotional activities at academic conferences and seminars attended by VEST members. Candidates had to be diagnosed with idiopathic or structural epilepsy by IVETF Tier II confidence level and were required to meet one of the following conditions:

A) Canine patients with epilepsy who do not respond (> 2 seizures per 3 months) to 3 of the 4 recommended and therapeutic doses of antiseizure medications (ASM) in veterinary medicine listed below: phenobarbital (PB), potassium bromide (KBr), zonisamide (ZNS), and levetiracetam (LEV).
   Maximum permissible dosage or serum concentration (trough) for each ASM:
   - PB (trough serum concentration): 30–35 μg/mL
   - KBr (trough serum concentration): 2.5–3.0 mg/mL
   - ZNS (trough serum concentration): 40–50 μg/mL
   - LEV (dosage): 40–60 mg/kg thrice daily

B) Epileptic canine patients with severe adverse effects of ASMs and difficulty maintaining QoL. Patients with epilepsy who are taking multiple ASMs such as PB, KBr, ZNS, or LEV to control seizures, but have difficulty in maintaining QoL due to severe or multiple adverse effects or the underlying disease(s), which are affected by ASMs, as well as patients in whom seizure frequency increases when ASMs are reduced.
   Examples:
   - Patients with difficulty in daily life due to constant sedation and severe ataxia
   - Patients with an increase in seizures when ASMs are reduced
   - Patients who want to reduce the dosage of ASMs because they have or are developing hepatic or renal damage caused by ASMs, but the reduction of dosage makes seizure control difficult

C) Drug-resistant cases from structural epilepsy without progressive disease; patients with structural epilepsy diagnosed by MRI and CSF examination who do not have any progressive disease such as brain tumor or encephalitis; and patients whose only treatment target is seizure control and who match the first two conditions (refractory to 3 out of 4 ASMs or reduced QoL due to ASMs).
   The following are examples of conditions that may be targeted:
   - Cortical dysplasia (polymicogyria, lissencephaly, schizencephaly, etc.)
   - Post-traumatic epilepsy due to ulegyria caused by a previous head injury
   - Patients who have undergone brain tumor resection surgery in the past and have no recurrence of the tumor but are left with intractable epilepsy
   - Patients diagnosed with idiopathic or infectious encephalitis and had been treated with suitable therapy and are in remission, with no progression of encephalitis for more than 1 year without any drugs for encephalitis other than ASMs, but with drug-resistant post-encephalitis epilepsy only

When the owner(s) or the primary veterinarian(s) of the candidate dog(s) expressed interest in participating in the clinical trial, the primary veterinarian referred the case to a VEST member. Once a referral was received, all VEST members reviewed and discussed the clinical data of the candidate to confirm the accuracy of the epilepsy diagnosis and to determine whether the case met the abovementioned condition; specifically, whether it was a true case with drug-resistant epilepsy.

Following this assessment, a VEST member informed the approved case's owner about the purpose and design of the study, the source of funding, and the necessities and procedures for presurgical evaluations by a member of VEST. The owner was then required to sign the form to participate in the clinical trial and to proceed with the presurgical evaluations, which were financially supported.

4. Presurgical Evaluations and Selection of Appropriate Surgical Procedure

Approved candidates for epilepsy surgery were required to undergo the following presurgical evaluations[1]:

1) 24-hour video seizure monitoring (either at home or during hospitalization) – if the previously submitted ictal videos did not capture seizures from onset to full recovery and/or did not include all observed seizure types
2) Scalp EEG under sedation
3) Structural MRI using a 3.0-Tesla machine, including 3D sequences, diffusion-weighted imaging, and diffusion tensor imaging

Once these data were collected, the VEST members held a second meeting to review the findings and determine the most appropriate surgical procedures for each candidate. If, during this discussion, it remained unclear whether the candidate had focal epilepsy suitable for resective surgery, or generalized epilepsy, or focal epilepsy with multiple foci or an unidentifiable focus that would suggest palliative surgery, additional presurgical evaluations were required. These included:

4) Long-term video-EEG monitoring
5) Long-term video-intracranial EEG monitoring

The algorithm for selecting appropriate surgical procedures, including cortical resection, ventrolateral temporal lobectomy, lesionectomy, multiple subpial transection, corpus callosotomy, and vagus nerve stimulation, has been described in detail elsewhere.[2]

Three cases that underwent rostral or second-stage total corpus callosotomy, as described in the main text, followed this process. These cases were also discussed by the VEST members, and corpus callosotomy or vagus nerve stimulation was suggested as the most appropriate surgical option.

5. Anesthesia for corpus callosotomy and perioperative medications

All cases were anesthetized with intravenous propofol for induction, followed by maintenance with either a continuous rate of infusion (CRI) of propofol (3–6 mg/kg/hr) or inhalation of isoflurane (1.5–2.0%) with oxygen. For intraoperative analgesia, a CRI of fentanyl (2–4 μg/kg/hr) or remifentanil (15–30 μg/kg/hr) was administered. During intraoperative electrocorticography recording, the propofol CRI was reduced in dosage, or the inhaled isoflurane was switched to sevoflurane while maintaining the fentanyl CRI. After surgery, the fentanyl CRI was tapered off within 12 hours and replaced with a fentanyl patch (25 μg/hr) for 3 days.

To reduce brain swelling during surgery, mannitol (0.5–1.0 g/kg) or a concentrated solution of glycerin and fructose (0.5–1.0 g/kg) was administered over 15 minutes, as needed (ranging from none to four times during the operation). Cefalexin (20 mg/kg IV) was administered every 2 hours intraoperatively as prophylactic antibiotic therapy and continued at the same dose every 12 hours for 2 weeks postoperatively.

All patients were monitored continuously (directly or via video) in the intensive care unit for 10–14 days postoperatively. If clinical signs of intracranial hypertension (e.g., decreased consciousness, visual deficits, circling, or aimless pacing) appeared, additional infusions of concentrated glycerin and fructose (0.5–1.0 g/kg over 15 minutes) were administered as needed.

Feeding and water intake typically resumed 24 hours after surgery. After the scalp sutures were removed, patients were discharged from the hospital. Antiseizure medications were continued at the same dosages as before surgery and maintained for 12 months during the follow-up period.

6. References

1. Hasegawa D. Diagnostic techniques to detect the epileptogenic zone: Pathophysiological and presurgical analysis of epilepsy in dogs and cats. *Vet J.* (2016) 215:64–75. doi: 10.1016/j.tvjl.2016.03.005
2. Hasegawa D, Saito M, Kitagawa M. Neurosurgery in canine epilepsy. *Vet J.* (2022) 285:105852. doi: 10.1016/j.tvjl.2022.105852
